# Population Health and Health Inequality Impacts of the National Abdominal Aortic Aneurysm Screening Programme (NAAASP) in England

**DOI:** 10.1177/0272989X251388481

**Published:** 2025-11-13

**Authors:** Shainur Premji, Simon M. Walker, James Koh, Matthew Glover, Michael J. Sweeting, Susan Griffin

**Affiliations:** Centre for Health Economics, University of York, York, UK; Centre for Health Economics, University of York, York, UK; Centre for Health Economics, University of York, York, UK; National Institute for Health and Care Excellence, City Tower, Manchester, UK; Surrey Health Economics Centre, School of Biosciences, Faculty of Health and Medical Sciences, University of Surrey, Guildford, UK; Department of Population Health Sciences, University of Leicester, Leicester, UK; Centre for Health Economics, University of York, York, UK

**Keywords:** inequality, deprivation, aortic aneurysm, screening, cost-effectiveness

## Abstract

**Purpose:**

We conducted a distributional cost-effectiveness analysis (DCEA) using routinely collected data to estimate the population health and health inequality impacts of the National Abdominal Aortic Aneurysm Screening Programme (NAAASP) in England.

**Methods:**

An existing discrete event simulation model of AAA screening was adapted to examine differences between socioeconomic groups defined by Index of Multiple Deprivation, obtained from an analysis of secondary data sources. We examined the distributional cost-effectiveness of being invited versus not invited at age 65 y to screen using a National Health Service perspective. Changes in inequality were valued using a measure of equally distributed equivalent health.

**Results:**

The net health benefits of population screening (317 quality-adjusted life-years [QALYs] gained) were disproportionately accounted for by the effects on those living in more advantaged areas. The NAAASP improved health on average compared with no screening, but the health opportunity cost of the programme exceeded the QALY gains for people living in the most deprived areas, resulting in a negative net health impact for this group (106 QALYs lost) that was driven by differences in the need for screening. Consequently, the NAAASP increased health inequality at the population level. Given current estimates for inequality aversion in England, screening for AAA remains the optimal strategy.

**Conclusion:**

Examination of the distributional cost-effectiveness of the NAAASP in England using routinely collected data revealed a tradeoff between total population health and health inequality. Study findings suggest that the NAAASP provides value for money despite health impacts being disseminated to those who are more advantaged.

**Highlights:**

- This study examines the population health and health inequality effects of the National Abdominal Aortic Aneurysm Screening Programme (NAAASP) between socioeconomic groups defined by Index of Multiple Deprivation.
- Findings suggest a tradeoff between total population health and health inequality.
- Given current estimates for inequality aversion in England, screening remains the optimal strategy relative to not screening.
- Opportunities remain to reduce inequality effects for those most vulnerable through targeted approaches.

Over the past several decades, both life and healthy life expectancies have increased across the population of England.^1^ Despite this, health inequalities have remained pervasive^2^; males born in 2020 in the most affluent areas of England are expected to live 10 y longer than males born in the least affluent areas.^1,3^ While health inequalities may be a result of systemic variations in the risk factors for disease, they may also be caused by structural differences in delivery, access, utilisation, and outcomes related to health policies and interventions.^4–7^ In England, the 2022 Health and Care Act details a requirement to tackle health inequality,^7^ and health decision makers have the dual objective of increasing population health while reducing unfair health inequalities.

Distributional cost-effectiveness analysis (DCEA) is a structured framework that estimates the total population health and health inequality effects of interventions and outlines the potential tradeoffs between each.^4^ Importantly, DCEA makes explicit the distribution of health opportunity costs, that is, the distribution of costs and consequences from relevant alternative investments to the intervention(s) being evaluated.^8^ The application of DCEA has been explored across various settings in health care.^4,9–15^ One area that remains relatively unexplored is in the use of secondary (routine) data sources to support DCEA. Secondary data sources, such as health care administrative records and population registries, are unique in that they are collected by health care providers, insurers, and other institutions for the purposes of the routine administration of health care and not primarily for medical research.^16^ Yet, these sources provide access to system performance data for large, representative populations, signifying its potential value in incorporating routine access, delivery, and utilisation information for different equity-relevant population subgroups within a structured DCEA. In this study we conduct a DCEA of a current National Health Service (NHS) policy: the National Abdominal Aortic Aneurysm Screening Programme (NAAASP) in England using routinely collected data. The NAAASP was chosen for review following consultation with policy stakeholders, and this work aligns with the programme’s own desire to address inequalities in screening.^16^ Results from this study can be used to support potential programme changes and improvements.

## Abdominal Aortic Aneurysm Screening

Abdominal aortic aneurysm (AAA) is a potentially life-threatening condition involving a permanent enlargement of the aorta as it passes through the abdomen.^17^ This predominantly asymptomatic condition, if left undetected, may result in sudden rupture and death for 3,000 to 4,000 individuals per year in England, approximately 0.01%.^18^ Established in 2009 across 6 early implementation sites, the NAAASP was fully available across England by 2013^19^ and involves a 1-time screen for men the year they turn 65 y of age. Eligible individuals are sent a letter inviting them to undergo an abdominal ultrasound scan.^18,20^ Those who attend and are found to have an enlarged aorta (3.0–5.4 cm) are offered routine surveillance, and those with an aortic diameter ≥5.5 cm are referred for further consultation and possible surgical intervention.^18,20^

Previous research has examined the cost-effectiveness of AAA screening in England and found that screening men at age 65 y would cost £6,352 per quality-adjusted life-year (QALY) gained, while screening women using the same protocol would cost £31,000 per QALY gained.^21–23^ However, there are inequalities associated with AAA and the NAAASP that have not been previously incorporated. For example, AAA is twice as common among the 10% of the population living in the most deprived areas versus the 10% living in the least deprived areas of England (odds ratio: 2.1, 95% confidence interval: 1.77–2.27), and men are 4 times more likely to be affected compared to women.^18,24^ Furthermore, screening uptake and attendance varies with local deprivation and geographic distance to a nearby city center.^18^ Given these inequalities in prevalence, access, and uptake of screening and treatment, the NAAASP presents an interesting case study to conduct a DCEA and aims to capture the combined effects resulting from structural differences in delivery, access, utilisation, and outcomes, the “staircase of inequality”,^9^ along the screening pathway, and to demonstrate the impact of being versus not being invited to screen on total population health and health inequality.

## Methods

### Model Overview

A previously developed and publicly available individual-level discrete event simulation (DES) model in the R programming language^21,23,25^ was updated and adapted in this study to examine differences in costs and outcomes across socioeconomic groups defined by Index of Multiple Deprivation (IMD). IMD is an area-based measure that ranks the deprivation level of small areas in England based on the proportion of people in each area that experience deprivation across 8 dimensions: income, employment, education, health, housing, living environment, crime, and access to services.^26^ For this study, IMD was divided into quintiles, where quintile 1 (Q1) represented 20% of the population living in the most deprived areas and quintile 5 (Q5) represented 20% of the population living in the least deprived areas of England.

The original model provided an estimate of the impact of the NAAASP compared with no NAAASP in terms of incremental cost per QALY gained by examining the average population costs and outcomes for women eligible for screening.^21,23^ The adapted model estimates costs and outcomes in subpopulations of men eligible for screening, differentiated by IMD. These estimates are then used to examine the impact of the NAAASP compared with no NAAASP on the socioeconomic distribution of population health. To achieve this, the DCEA combines information from the adapted model with information about the socioeconomic distribution of quality-adjusted life expectancy (QALE) in England^27^ and the health opportunity cost of NHS expenditure on subpopulations defined by IMD.^28^ This distribution of opportunity cost represents the health that is forgone by each subgroup as additional costs are imposed on the NHS budget and other health care interventions are displaced. Intervention effects are described in terms of whether the NAAASP increases the mean population net health benefit, measured in QALYs, and whether it reduces differences in QALE between subpopulations defined according to IMD.

Full details on the original model are provided elsewhere,^21,23^ and the model structure can be viewed in Supplementary Appendix 1. Briefly: the model was initiated at the time of invitation to screen (age 65 y) and ended upon death. Each individual invited to screen had an identical simulated counterpart in terms of age, aortic diameter at baseline, rate of aortic growth, and potential time of death^23^ who was not invited to screen. The model characterises the joint continuous AAA growth and rupture risk and allows for variability in the clinical pathway; variations included dropout from surveillance, incidental detection of AAA, referral for AAA repair consultation, uptake of surgical intervention, and waiting times for consultation and elective surgery, allowing for differences by IMD.^23^ The adapted model simulated key screening and clinical events for 5 million men (1 million men per IMD) over a lifetime time horizon, using an NHS health and personal social services perspective.

### Data Sources

The original model used data from a variety of randomised controlled trials (RCTs) and observational data sources and had been validated against data for men from the Multicentre Aneurysm Screening Study (MASS) trial.^22^ For the current study, we revisited these data sources to conduct additional analyses to reflect differences in screening access, uptake, and outcomes between IMD groups where data were available. We obtained routinely collected registry data between 2009 and 2019 from the NAAASP and the National Vascular Registry (NVR) and updated the associated model parameters to account for differences between IMD groups. The NAAASP extracts data on key performance indicators from the Screening Management and Referral Tracking (SMaRT) national information technology system and provides operational information on the screening programme (e.g., demographic, appointment, screening, referral, and outcomes data from across England).^29,30^ The NVR conducts routine mandatory clinical audits for the quality of care provided by NHS vascular units and reports on benchmarked process and outcome indicators for patients undergoing AAA repair across the United Kingdom.^31^ Parameters in the original model that were not updated nor able to be differentiated by IMD included those sourced from RCTs that did not report a breakdown by IMD.^32,33^ Following data linkage across multiple NAAASP data tables, cleaning, and application of the eligibility criteria (males ≥64 y of age), the final sample size in the NAAASP data consisted of 2,007,414 unique individuals with 3,525,322 recorded appointments between 2009 and 2019; in NVR, the final sample size consisted of 27,335 unique individuals over the same period. Given the NAAASP data reported IMD in decile groups, whereas the NVR reported IMD in quintile groups, we combined decile groups as required to derive the IMD quintile groups for this study.

### Updated Parameters

Updated model parameters included the distribution of aortic sizes at age 65 y; various screening, attendance, dropout from surveillance, and type of AAA repair characteristics; and mortality outcomes, as reported in Table 1. The data from the NAAASP and NVR were used to inform eligibility, access, uptake, and outcome parameters for those invited to screen, and to inform the simulation of eligible men who were not invited to screen, who shared the same key IMD-specific (e.g., aortic diameter at baseline, emergency versus elective repair, potential time of non–AAA-related death) and non–IMD-specific (e.g., age, incidental detection rate, rate of aortic growth) characteristics (Table 1). While we captured variation in AAA incidence, baseline aortic diameter, and the screening pathway, we assumed no variation in the effect of follow-up treatment. We also assumed no differences in data quality or reporting across time. Updated model parameters adhered to the definitions and methods provided in the original study,^21^ unless described here.

**Table 1:** Updated Screening, Attendance, Surveillance, and Treatment Model Parameters

Item	Source	Value: Q1 Most Deprived	Value: Q2	Value: Q3	Value: Q4	Value: Q5 Least Deprived
Baseline aortic diameter	NAAASP	IMD-specific distributions				
Proportion that required reinvitation to screening	NAAASP	0.3609	0.303	0.2505	0.2166	0.1865
Proportion that attends screening	Jacomelli et al.^18^	0.6736	0.7403	0.7895	0.8157	0.8342^a^
Proportion nonvisualised	NAAASP	0.0187	0.015	0.0117	0.0104	0.009^a^
Proportion dropped out from surveillance	NAAASP	0.0481	0.0395	0.0381^a^	0.0407	0.0415
Mean waiting time to consultation (y)	NAAASP	0.0538	0.0605	0.0473	0.0413^a^	0.0515
Mean waiting time to elective surgery (y)	NAAASP	0.3445	0.3461	0.2847^a^	0.2980	0.3263
Proportion receiving elective open versus EVAR^b^	NVR	5.53 − 0.11 × age +0.03 × aorta size	6.22 − 0.11 × age − 0.02 × aorta size	5.92 −0.11 × age + 0.02 × aorta size	7.09 − 0.12 × age + 0.02 × aorta size^a^	6.67 − 0.12 × age + 0.02 × aorta size
Odds of elective EVAR 30-d operative mortality^b,c^	NVR	−3.59 − 0.03 × age + 0.01 × aorta size				
Odds of elective open repair 30-d operative mortality^b,c^	NVR	−4.11 − 0.07 × age + 0.03 × aorta size				
Odds of receiving emergency open versus EVAR^b^	NVR	2.06 − 0.02 × age	3.50 − 0.04 × age	3.99 − 0.047 × age	2.35 − 0.025 × age	0.73 − 0.004 × age^a^
Odds of emergency EVAR 30-d operative mortality^b,c^	NVR	−5.60 + 0.02 × age				
Odds of emergency open repair 30-d operative mortality^b,c^	NVR	−17.68 + 0.18 × age				
Non-AAA mortality rate	ONS	IMD-specific distributions				

AAA, abdominal aortic aneurysm; EVAR, endovascular aneurysm repair; IMD, Index of Multiple Deprivation; NAAASP, National AAA Screening Programme; NHS, National Health Service; NVR, National Vascular Registry; ONS, Office for National Statistics; Q, population quintile.

a Best-case values used to represent leveling-up scenario analysis.

b Coefficients represent the log odds, derived using logistic regression models with age and/or aorta size as model covariates.

c Due to insufficient sample sizes, we were unable to obtain IMD-specific estimates.

### Non-AAA Mortality Rate

To derive the non-AAA mortality rate for each IMD quintile, we obtained the AAA age- and sex-specific number of deaths using data from the Office for National Statistics (ONS).^34^ As these data were available for only the population of England and Wales combined, we assume they are representative of the population in England. AAA was identified using the International Classification for Disease ICD-10 code I71 (“aortic aneurysm and dissection”). Using the proportion of the age- and sex-specific population for each IMD decile provided in ONS population estimates for England,^35^ we estimated the age-, sex-, and IMD-specific AAA number of deaths and subtracted this from the ONS population estimates for all-cause number of deaths.^34^ Finally, we followed the ONS national life table approach^36^ to derive the age-, sex-, and IMD-specific non-AAA mortality rate, which was inputted into the adapted model as an age-based distribution for each IMD.

### Health-Related Quality of Life

Age-, sex-, and IMD-adjusted health-related quality of life (HRQoL) was sourced from English population-level survey data using a previously published study.^27^ In line with the original model,^23^ we assumed that the invitation to screen for AAA did not affect individuals’ HRQoL, an assumption supported by a recent systematic review.^37^

### Costs

Screening, consultation, monitoring, and repair costs (Table 2) were updated from 2014/2015 to 2020/2021 British pound sterling using the NHS cost inflation index.^38^

**Table 2:** Updated Costs

Item	Source	2020/2021 Price
Invitation to screen	NAAASP	£2.13
Reinvitation	NAAASP	£2.13
Screening scan	NAAASP	£40.43
Surveillance scan	NAAASP	£85.37
Surveillance scan after turn down for surgery	Expert opinion, NHS reference costs	£85.37
Consultation for elective surgery	MASS, NHS reference costs	£352.41
Elective EVAR	IMPROVE RCT, HES, NHS reference costs	£15,645.68
Elective open repair	IMPROVE RCT, HES, NHS reference costs	£14,732.84
Emergency EVAR	IMPROVE RCT, HES, NHS reference costs	£22,283.91
Emergency open repair	IMPROVE RCT, HES, NHS reference costs	£22,608.57
Reintervention after elective EVAR	EVAR-1 RCT	£10,253.39
Reintervention after elective open repair	EVAR-1 RCT	£13,687.78
Reintervention after emergency EVAR	EVAR-1 RCT	£10,253.39
Reintervention after emergency open repair	EVAR-1 RCT	£13,687.78
Surveillance after EVAR operation	Expert opinion, NHS reference costs	£298.84
Surveillance after open repair	Expert opinion, NHS reference costs	£211.02

AAA, abdominal aortic aneurysm; EVAR, endovascular aneurysm repair; EVAR-1, UK Endovascular Aneurysm Repair Trial 1; IMPROVE, Immediate Management of Patients with Rupture: Open Versus Endovascular repair; MASS, Multicentre Aneurysm Screening Study; NAAASP, National AAA Screening Programme; NHS, National Health Service; NVR, National Vascular Registry; RCT, randomised controlled trial.

### Outcomes

The model outcomes include health care costs ( $c_{i,t}$ ) and QALYs ( $q_{i,t}$ ) by IMD quintile ( i=1,2,3,4,5 ) for individuals targeted by the NAAASP ( t ) in the presence of the policy ( t=1 ) and in the absence of the policy ( t=0 ). All costs and outcomes were discounted at an annual rate of 3.5%, in line with UK guidelines.^39^ Model outcomes were used to estimate the total health and health inequality effects at the population level, as described below and using the notation provided in Table 3.

**Table 3:** Distributional Cost-Effectiveness Analysis Notation.

Notation	Definition
N	Total population size
ρ(*t*)_*i*_	The proportion of subgroup i who are targeted by the health care policy
$n_{i}$	The number of people in subgroup i
$c_{i.t}|t=1$	The lifetime health care cost for an individual in subgroup i targeted by the health care policy, in the presence of the health care policy
$c_{i.t}|t=0$	The lifetime health care cost for an individual in subgroup i targeted by the health care policy, in the absence of the health care policy
$q_{i.t}|t=1$	The lifetime QALYs for an individual in subgroup i targeted by the health care policy, in the presence of the health care policy
$q_{i.t}|t=0$	The lifetime QALYs for an individual in subgroup i targeted by the health care policy, in the absence of the health care policy
$\mathrm{nh}b_{i}$	Net health benefit from the health care policy for an individual in subgroup i
$h_{i}|t=1$	The quality adjusted life expectancy at birth of an individual in subgroup i, in the presence of the health care policy
$h_{i}|t=0$	The quality-adjusted life expectancy at birth of an individual in subgroup i, in the absence of the health care policy
$\rho {\left(k\right)}_{i}$	The proportion of the marginal product of health expenditure received by subgroup i
k	The marginal product of health expenditure
HOC	Total population health opportunity cost from a decision to implement the health care policy
$H_{\mathrm{EDE}}$	Population equally distributed equivalent health

EDE, equally distributed equivalent; QALY, quality-adjusted life-year.

### DCEA

The total population in England, N ; the number of individuals living in each IMD quintile, $n_{i}$ ; and the proportion of the English population in each IMD quintile who are males aged 65 y and thus eligible for the NAAASP, $\rho {\left(t\right)}_{i}$ , were provided through ONS population tables^35^ and reported in Supplementary Appendix 2. Of all 65-y-old men eligible for screening, 17% lived in the most deprived quintile and 22% lived in the least deprived quintile. The health opportunity costs of any change in health care resource use imposed by the decision to screen are borne across the whole of the English population.

### Health Opportunity Cost

The total health opportunity cost was derived by multiplying the total incremental cost of invitation (compared with no invitation) to screen by the average marginal productivity of health expenditure, k , based on an empirical study of the marginal rate at which current NHS expenditure produces QALYs and estimated at 1 additional QALY per £13,000,^40^ as shown in equation 1.

(1) $$\mathrm{HOC}=k\sum_{1}^{5}\{[\left(c_{i,t}|t=1\right)-\left(c_{i.t}|t=0\right)]\rho {\left(t\right)}_{i}n_{i}\}$$

This health opportunity cost represents how many QALYs could have been generated by an alternative use of resources. The socioeconomic distribution of the health opportunity cost (i.e., the proportion of the marginal product that would have benefited individuals in subgroup *i*, $\rho {\left(k\right)}_{i}$ ) was informed by a previous study.^28^ This study estimated that those living in the most deprived quintile (Q1) accrued 26% of the marginal effects, whereas those in the least deprived quintile (Q5) accrued 14% of the marginal effects.

### Population Health with the NAAASP

The distribution of QALE at birth for the population by IMD, $h_{i}$ , was also obtained from a previous study.^27^ This is assumed to be the distribution of health in the presence of the current NAAASP policy, $h_{i}|t=1$ . The adapted model estimates the difference in net health benefits (in terms of QALYs) with the NAAASP relative to no screening programme for each IMD quintile at the population level, as shown in equation 2.

(2) $$\mathrm{nh}b_{i}=\frac{1}{n_{i}}\{\left[\left(q_{i.t}|t=1\right)-\left(q_{i.t}|t=0\right)\right]\rho {\left(t\right)}_{i}n_{i}-\rho {\left(k\right)}_{i}\mathrm{HOC}\}$$

### Population Health without the NAAASP

We subtract these net health benefits from the baseline distribution of QALE to represent the population distribution of QALE by IMD that would be expected without the presence of the NAAASP, as shown in equation 3.

(3) $$(h_{i}|t=0)=\left(h_{i}|t=1\right)-\mathrm{nh}b_{i}$$

### Inequality Effects

To measure the extent of inequality in the population distribution of health by IMD, we assess the gap in QALE between the most advantaged and least advantaged quintile with and without the invitation to screen. We also calculate an Atkinson index^41^ to determine whether the NAAASP increases or decreases relative inequality in QALE by IMD compared to no screening programme. The Atkinson index is a measure of social welfare and social welfare–based inequality, in which the social value of inequality effects is determined by an inequality aversion parameter, ε. The higher the value of ε, which can range from zero to infinity, the greater the public’s aversion to inequality. We calculate the Atkinson index for a range of inequality aversion parameter values (0 ≥ε≤ 20) and report the main results using an estimate for income-related health inequality aversion for England of ε = 3.5.^42^ To express the value of the change in inequality in terms of QALYs, we use the Atkinson social welfare function to compute the equally distributed equivalent QALE (H_EDE_) for the estimated population distributions of QALE by IMD.

(4)^ i
^ $$H_{\mathrm{EDE}}=N{\left[\frac{1}{N}\sum_{1}^{5}n_{i}h_{i}^{1-\varepsilon }\right]}^{\frac{1}{1-\varepsilon }}$$

The change in equally distributed equivalent (EDE) QALE with invitation compared with no invitation to screen $\left(H_{\mathrm{EDE}}\left|t=1)-(H_{\mathrm{EDE}}\right|t=0\right)$ is used to indicate the social value of the NAAASP in terms of QALYs. EDE is improved by increases in overall health and (in the presence of ε > 0) by a reduction in inequality. To isolate the value of the NAAASP impact on inequality alone, we subtract from the change in EDE the total net population health, $\sum_{1}^{5}\mathrm{nh}b_{i}n_{i}$ , with the NAAASP versus without. This difference is used to represent the proportion of the change in EDE with the NAAASP compared with no screening programme that is accounted for by a change in inequality. We plot the QALY value of the change in net population health and the QALY value of the change in health inequality on the equity-efficiency impact plane to illustrate the results. We also evaluate the threshold value of inequality aversion parameter at which the social value of inviting versus not inviting men to screen would be considered distributionally equivalent, $\left(H_{\mathrm{EDE}}\left|t=1)=\;(H_{\mathrm{EDE}}\right|t=0\right)$ .

### Scenario Analyses

We conducted 2 scenario analyses. First, to explore potential areas of uncertainty in our findings, we examined model results in which the health opportunity costs were apportioned equally across IMD quintiles, as opposed to our base case, in which these health opportunity costs were distributed in a manner that disproportionately favors those living in the more deprived areas of England. Second, to demonstrate the potential social benefits that would result from removing inequalities related to screening implementation, we examined a “leveling-up” NAAASP policy, in which we assumed implementation of an “optimal” one-off screening. This involved setting access and uptake parameters to the best-case value presented from all IMD groups, as indicated in Table 1. Note that parameters related to eligibility and associated costs were not adjusted to support this scenario; each IMD quintile was provided equivalent “best-case” access or delivery to the programme only.

## Results

**Figure 1:**
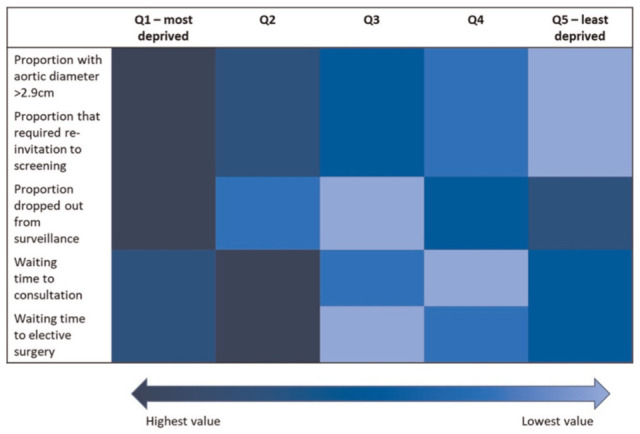
Staircase of inequality along the AAA screening pathway.

Figure 1 demonstrates the differences by IMD for parameters related to the need, uptake, and delivery of the screening programme. Individuals living in the most deprived areas of England (Q1) had the highest need for screening, as they were most at risk of AAA rupture. These individuals also had low uptake of the screening programme, as they were the highest proportion that required reinvitation to the screening programme and the highest proportion to drop out of surveillance. Individuals in Q1 further experienced long waiting times for consultation and elective surgery, indicating further inequality in the delivery of the screening programme for these areas of England. Individuals in Q2 were also at high risk of AAA rupture, but these individuals were less likely to drop out of surveillance relative to those in Q1. Relative to those in Q1, individuals in Q2 experienced longer waiting times for consultation and elective surgery. Those in Q3 had a moderate need for screening, were least likely to drop out of surveillance, and experienced short waiting times for consultation and elective surgery. Those in Q4 had a lower need for screening compared with those living in more deprived areas, and a moderate proportion of those in Q4 dropped out of surveillance. Similar to Q3, these individuals experienced short waiting times for consultation and elective surgery. Finally, those living in the least deprived areas of England (Q5) experienced the lowest need for screening, as they were least at risk of AAA rupture, but a high proportion of individuals dropped out of surveillance. These individuals experienced moderate waiting times for consultation and elective surgery.

### Model Results

The results presented in Table 4 provide the total and IMD-specific average incremental costs and health effects. The incremental costs of being invited relative to not invited to screen were highest for those in Q2 (£64.65 per individual) and lowest for those in Q5 (£58.91 per individual). The incremental health effects were greatest among Q3 (0.0064 QALYs gained per individual) and lowest among those most deprived in Q1 (0.0052 QALYs gained per individual).

**Table 4:** Simulated Results.

	Individual Level		Population-Level Effect of the NAAASP versus No NAAASP				
	Incremental Costs (£) $\left(c_{i,t}|t=1\right)-\left(c_{i.t}|t=0\right)$	Incremental QALYs $\left(q_{i,t}|t=1\right)-\left(q_{i.t}|t=0\right)$	% of 65-y-Olds (Male Population)	Total Cost (£ Millions)	Total QALYs	Total HOC (QALY)	Net Health Impact (QALY)
Q1: most deprived	£61.29	0.0052	17%	£2,869,500	244	350	−106
Q2	£64.65	0.0062	19%	£3,377,577	323	291	32
Q3	£63.59	0.0064	21%	£3,717,354	374	288	86
Q4	£59.84	0.0054	22%	£3,674,595	331	212	120
Q5: least deprived	£58.91	0.0061	22%	£3,585,851	370	185	185
Total	—	—	100%	£17,224,876	1,642	1,325	317

HOC, health opportunity cost; NAAASP, National Abdominal Aortic Aneurysm Screening Programme; Q, quintile; QALY, quality-adjusted life-year; QALE, quality-adjusted life expectancy.

Once individual-level effects were aggregated to reflect the total number of men aged 65 y across each IMD subgroup, the direct health gain was highest for those in Q3 (374 QALYs gained) and lowest for those in Q1 (244 QALYs gained), while the total costs were highest for those in Q3 (£3.7 million) and lowest for those in Q1 (£2.9 million). Overall, inviting men to screen for AAA generated 1,642 QALYs.

The opportunity cost of implementing the screening programme for AAA falls across the whole population of England, with the potential to use the resources to fund many other health care interventions. In total, we estimate an opportunity cost of 1,325 QALYs at the population level, resulting from the additional resource use implications of the programme. Those in the most deprived quintile (Q1) bear the greatest opportunity cost (350 QALYs lost; Table 4), while those in the least deprived quintile (Q5) bear the lowest opportunity cost (185 QALYs lost).

Upon adjusting the total incremental health impact of screening by the total opportunity cost (Table 4), those in the least deprived quintile, Q5, experienced the greatest incremental net health benefits from the introduction of a one-off screen at 65 y old (185 QALYs gained). Those in the most deprived quintile, Q1, experienced a loss in incremental net health (106 QALYs) from the introduction of the NAAASP.

**Figure 2:**
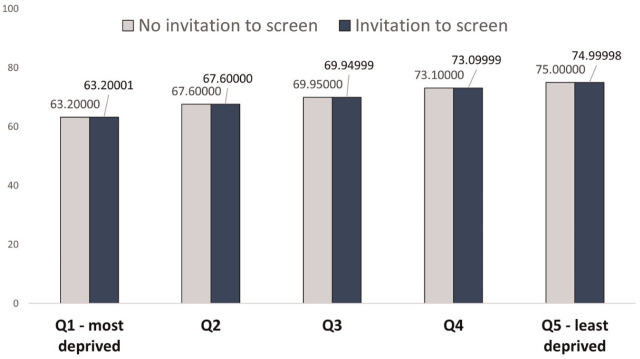
Average individual quality-adjusted life expectancy.

In Figure 2, the baseline QALE for men at age 65 y is represented by those who are invited to screen for AAA, whereas the QALE for those not invited to screen is represented by the baseline QALE minus the incremental health impact for the population in QALYs. Near-equivalent differences can be seen in Figure 2 across all IMD quintiles, and disparities between IMD are largely driven by baseline QALE.

### Evaluating the Equity-Efficiency Impact

**Figure 3:**
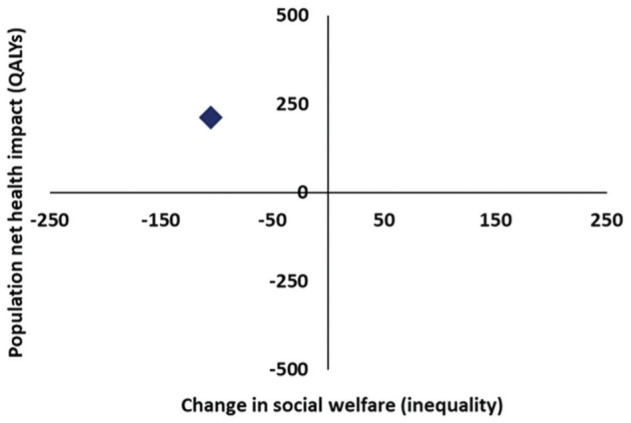
Equity-efficiency impact plane. QALY, quality-adjusted life year.

Screening for AAA fell within the northwest quadrant of the equity-efficiency impact plane (Figure 3), suggesting that while inviting men to screen for AAA improved total net population health (317 QALYs, where 423 QALYs were gained for those in Q2–Q5), it also increased unfair health inequality in England, where 106 QALYs were lost for those in Q1.

**Figure 4:**
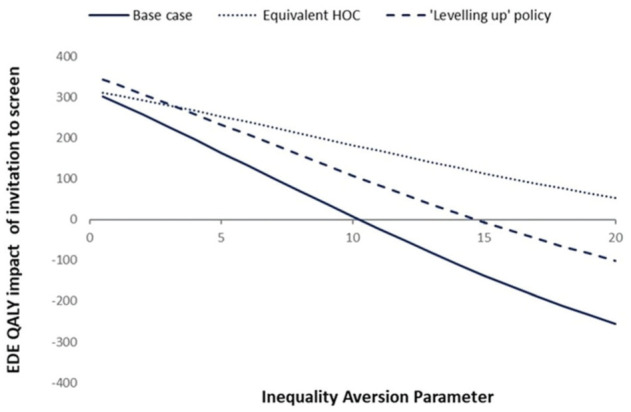
Equity-weighted population health impact of the National Abdominal Aortic Aneurysm Screening Programme. EDE, equally distributed equivalent; HOC, health opportunity cost; QALY, quality-adjusted life years.

Using public preferences for inequality aversion, ε=3.5 (our base case), Figure 4 suggests that inviting men aged 65 y to screen for AAA remains the preferred strategy. Figure 4 further suggests that the point at which our alternative policies to invite, versus not invite, men to screen for AAA would be considered equivalent is at ε = 10.

### Scenario Analyses

A scenario analysis in which the health opportunity costs of screening were allocated equally across IMD quintiles (“equivalent HOC”) resulted in similar findings, where those in Q1 still experienced a loss in incremental net health but to a lesser extent (21 QALYs lost; see Supplementary Appendix 3). Under this scenario, there is no value of inequality aversion up to ε = 20 for which no screening programme is preferred over the NAAASP (Figure 4).

Under the leveling-up NAAASP policy, in which we assumed one-off screening was optimised for all individuals, results were also similar, in which those in Q1 experienced a loss of 65 QALYs (Supplementary Appendix 4), compared with 106 QALYs lost in our base case. Under this situation, a value of inequality aversion of ε > 15 would result in a preferred policy of no screening for AAA, as shown in Figure 4.

## Discussion

This study aimed to conduct a DCEA to demonstrate the value for money and inequality effects of a population-based screening programme in the NHS. The NHS provides primary, secondary, and tertiary health care services for free at the point of care, is available universally, and is funded through national taxpayer contributions.^43^ In this study, DCEA provided a useful framework to evaluate population-based AAA screening in terms of its impact on total health and health inequality across different equity-relevant subgroups of the population, relative to no screening. As DCEA was only recently introduced within the field of health economics,^9,11^ its feasibility across different contexts and settings continues to be explored.^12–15,44^

In adapting the model for DCEA, we found evidence for differences by IMD across multiple parameters along the staircase of inequality^9^ in the NAAASP. Our analysis captures the combined effects for each IMD, allowing an overall assessment of equity in policy impacts. Previous analyses using DCEA methods have been unable to capture the combined effects of inequality related to variations in the risk of disease (health-related variations) and structural differences in the access and uptake of programmes and services designed to improve population health (health care–related variations).

Our findings suggest that the NAAASP improved average population health while increasing health inequality in England. Some of the inequality could be attributed to health-related variations across groups (e.g., individuals are required to live long enough to be eligible for screening, uptake, and delivery). While those in the most deprived quintile (Q1) had the greatest need for screening, as demonstrated by the large proportion of those with an enlarged abdominal aorta, these individuals had low uptake of the programme, and a high proportion required a reinvitation to screen. Those who participated in screening experienced long waiting times for consultation and elective surgery, indicating (health care-related) inequalities in programme delivery. These differences ultimately resulted in this group incurring the lowest incremental health impact per individual (0.0052 QALYs). The younger age distribution in the most deprived area also means that this group has the smallest number of individuals who lived long enough to be eligible for screening; consequently, they accumulated the smallest total incremental health benefit of 244 QALYs gained. Once we factored in the health opportunity costs, the incremental net health impact for those in the most deprived quintile was negative, indicating a loss in average population health of 106 QALYs. On the other hand, those in Q3 had a moderate need for screening, were least likely to drop out of surveillance, and faced short waiting times for consultation and elective surgery. Our findings indicated this group accumulated the greatest individual and total incremental health benefits (0.0064 and 374 QALYs gained, respectively). The contrast between these 2 groups exemplifies one of the benefits of conducting a DCEA, which is providing greater transparency of the distributional effects across subgroups of the general population.^11^

At a population level, determining the best policy option (screening versus not screening for AAA) requires knowledge of how each option would change both the total health benefits and the distribution of health-related outcomes.^9^ According to our findings, screening for AAA results in greater total population health (1,642 QALYs) relative to not screening (1,325 QALYs; Table 4). However, screening through the NAAASP also results in greater inequality at the population level compared with not screening, indicating there is a tradeoff that needs to be made between the dual policy objectives of maximising total population health and reducing health inequalities. DCEA provides a framework within which these aggregate and distributional tradeoffs can be examined, with an overall view of whether the tradeoff being made is worthwhile. This approach extends traditional cost-effectiveness analyses, which are concerned with maximising population health, and follows a utilitarian approach.^45,46^

Theories of distributive justice are concerned with the allocation of societal goods and resources in a manner considered to fairly increase welfare.^47^ Rawlsian prioritarianism and egalitarianism offer alternative frameworks to the utilitarian approach for evaluating the distribution of resources.^47^ These approaches argue that the appropriateness of a conventional utilitarian focus on maximising collective welfare in resource allocation relies on all individuals having equal access to opportunities, which they do not.^45,48^ Further, these theories acknowledge that disparities in opportunity arise from factors both within and outside an individual’s control, and accordingly, they incorporate aversions to inequality to differing extents.^45,49^

While Rawlsian prioritarianism posits that social welfare orderings should rank prioritise the outcomes of those who are worse off, irrespective of the origins of their disadvantage,^45,48^ the egalitarian, or equality of opportunity, perspective selectively favors disadvantages that arise from circumstances beyond an individual’s own control, rather than those attributable to factors within their control.^45^ To adopt the egalitarian perspective would then require additional information on the sources of inequality and the degree of individual responsibility within these sources—endorsing a “nonwelfarist” approach to social justice.^45^

For the purposes of determining whether the increase in inequality experienced through the NAAASP is an acceptable tradeoff to make in relation to the overall gain in population health, this study undertook a Rawlsian prioritarian approach and adopted the most recent estimate that is available to represent the level of aversion to health inequality in England (ε = 3.5).^42^ Using this value, Figure 4 demonstrates that health-related EDE is improved, implying there is still positive social value of the NAAASP. This suggests that the current exacerbation in health inequality from AAA screening at the population level is not sufficient to warrant a change in policy. If inequality aversion were to tip above ε = 10, however, this would indicate that the tradeoff being made between overall population health and health inequality would be considered socially unacceptable and instead would warrant a change in policy (Figure 4).

Our findings show that those in Q1 also experienced health care–related inequalities, where they may not be participating in the programme despite a high need for screening. This suggests there may be an opportunity to target policies for those in Q1. Areas for future research include understanding why individuals in this group are most likely to drop out of surveillance and why they experience long waiting times for consultation and elective surgery. Notably, our leveling-up scenario provided an indication that even when health care–related inequalities are addressed, inequality will still increase at the population level, albeit to a lesser extent than is currently observed (65 vs 106 QALYs lost), largely due to differences in the need for screening, or health-related variations, among this group. Evolving evidence suggests the importance of more upstream health-related interventions, such as those aimed at reducing smoking prevalence, which is associated with the growth rate of AAA.^50,51^

### Strengths and Limitations

A key strength of this study was our ability to use routine data to capture the combined effects of inequality across multiple parameters representing the need, uptake, and delivery of screening. Yet, it should be noted that not all parameters of interest were varied by IMD, particularly those obtained from RCTs. Therefore, there is a possibility that we have only partially captured the inequality effects of screening.^52^ On the other hand, emerging empirical analysis suggests there is value in prioritising which parameters are updated during model adaptation.^53^ On this basis, the current study updated distributional parameters for 3 of the top 4 model parameters identified as influencing cost-effectiveness decisions in previous 1-way sensitivity analyses.^23^ Another key strength in this study included our ability to explicitly model the tradeoffs between total population health and health inequality effects associated with the NAAASP.

Key limitations included those related to the use of routine data sources for this study. For example, there is a potential for misclassification bias arising from coding and/or data entry errors in the NAAASP and NVR data registries. This error is likely to be nondifferential, however, suggesting minimal effects on this analysis. Our model uses the observed past incidence of AAA and does not project any potential epidemiological or demographic changes. There were also differences in how we calculated some parameters for the DES model relative to the original study.^21,23^ Where possible, we conducted face validity checks of our parameters against those derived by the original study team to ensure consistency. However, we were unable to validate any simulated outcomes against the published literature or national data before or after NAAASP programme implementation. An additional limitation includes our inability to estimate the impact of uncertainty in model parameters using probabilistic sensitivity analysis. Furthermore, our chosen outcome measure of expected QALE at birth was more likely to find inequality increases in policies targeted at older age groups. For example, the QALE at birth in the most deprived (Q1) group is 63 y, and the NAAASP is provided conditional on survival to age 65 y. Similarly, our use of the QALE at birth indicator to measure distributional effects may not be the preferred choice of decision makers. However, in the absence of guidance on preferred measures from relevant stakeholders, we considered lifetime health to be a reasonable choice.

## Conclusion

This study demonstrated that routinely collected data effectively supported DCEA analysis of the NAAASP by enabling us to capture the staircase of inequality along the AAA screening pathway. While the total health benefits of population screening were disproportionately accounted for by effects on those more advantaged, current estimates for inequality aversion indicate that screening for AAA remains the optimal strategy. Nonetheless, there remain opportunities to reduce inequality effects by incentivising and rewarding participation and engagement in screening for those most vulnerable.

## Supplemental Material

sj-docx-1-mdm-10.1177_0272989X251388481 – Supplemental material for Population Health and Health Inequality Impacts of the National Abdominal Aortic Aneurysm Screening Programme (NAAASP) in England Supplemental material, sj-docx-1-mdm-10.1177_0272989X251388481 for Population Health and Health Inequality Impacts of the National Abdominal Aortic Aneurysm Screening Programme (NAAASP) in England by Shainur Premji, Simon M. Walker, James Koh, Matthew Glover, Michael J. Sweeting and Susan Griffin in Medical Decision Making

## Notes

i. For ε = 1, H_EDE_ = [Π_*i*_(*h_i_*)^1/N^] ×*N.*
