# Supplementary material for: Population Health and Health Inequality Impacts of the National Abdominal Aortic Aneurysm Screening Programme (NAAASP) in England
Source: Med Decis Making. 2025 Nov 13;46(2):202–15. doi: 10.1177/0272989X251388481 (PMC12769921; doi:10.1177/0272989X251388481)
Supplement: sj-docx-1-mdm-10.1177_0272989X251388481 – Supplemental material for Population Health and Health Inequality Impacts of the National Abdominal Aortic Aneurysm Screening Programme (NAAASP) in England [file sj-docx-1-mdm-10.1177_0272989X251388481.docx]

**Supplementary Appendix 1**

DES model structure


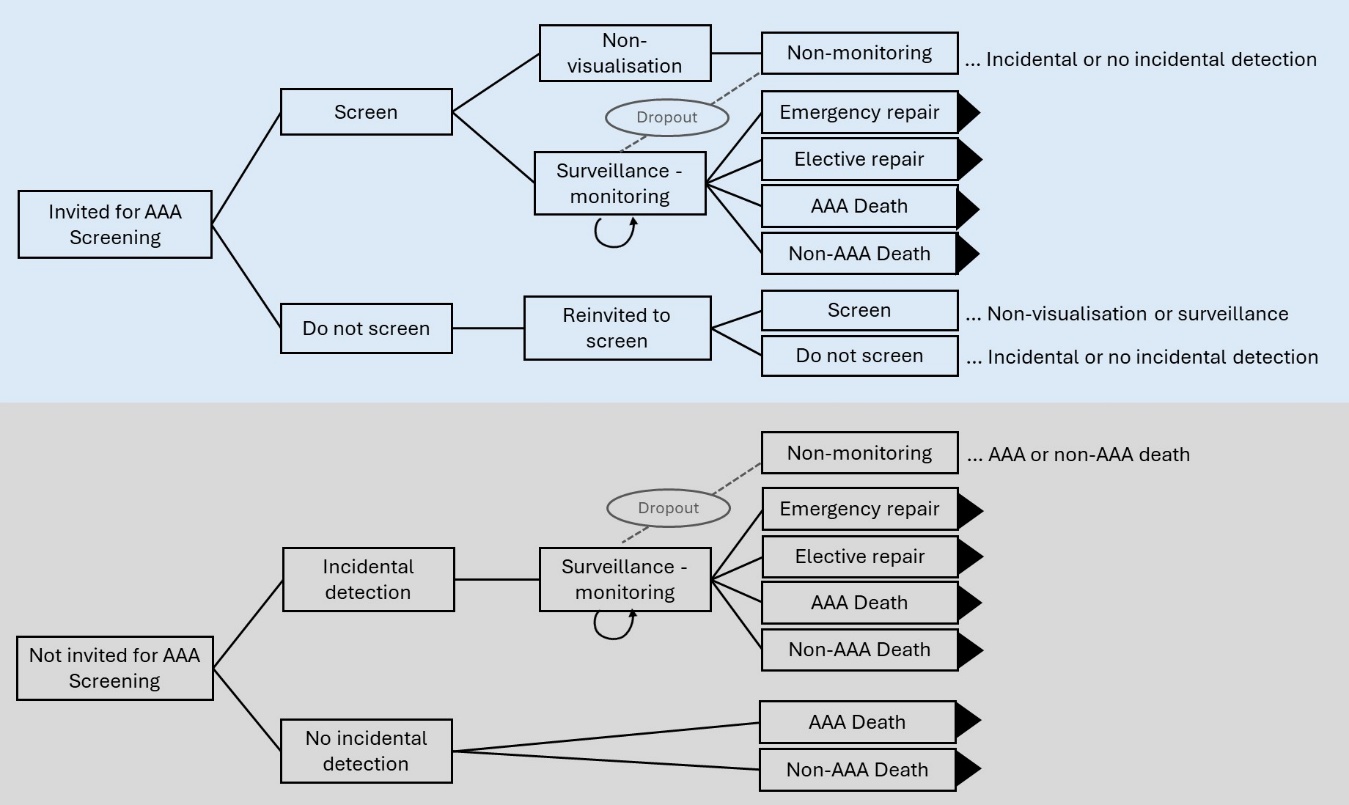


**Supplementary Appendix 2**

Population breakdown by IMD subgroup.^2^

| $N$ | Total population size in England | *N* = 56,286,961 |
| --- | --- | --- |
| $n_{i}$ | The number of people in each IMD subgroup | $n_{1}=$ 11,265,530  $n_{2}=$ 11,576,562  $n_{3}=$ 11,426,093  $n_{4}=$ 11,117,694  $n_{5}=$ 10,901,082 |
| 𝜌(𝑡)_𝑖_ | The proportion of each IMD subgroup that are targeted by the NAAASP | 𝜌(𝑡)_1_ = 0.42%  𝜌(𝑡)_2_ = 0.45%  𝜌(𝑡)_3_ = 0.51%  𝜌(𝑡)_4_ = 0.55%  𝜌(𝑡)_5_ = 0.56% |

**Supplementary Appendix 3**

Results of scenario analysis where opportunity costs were allocated equally across IMD groups.


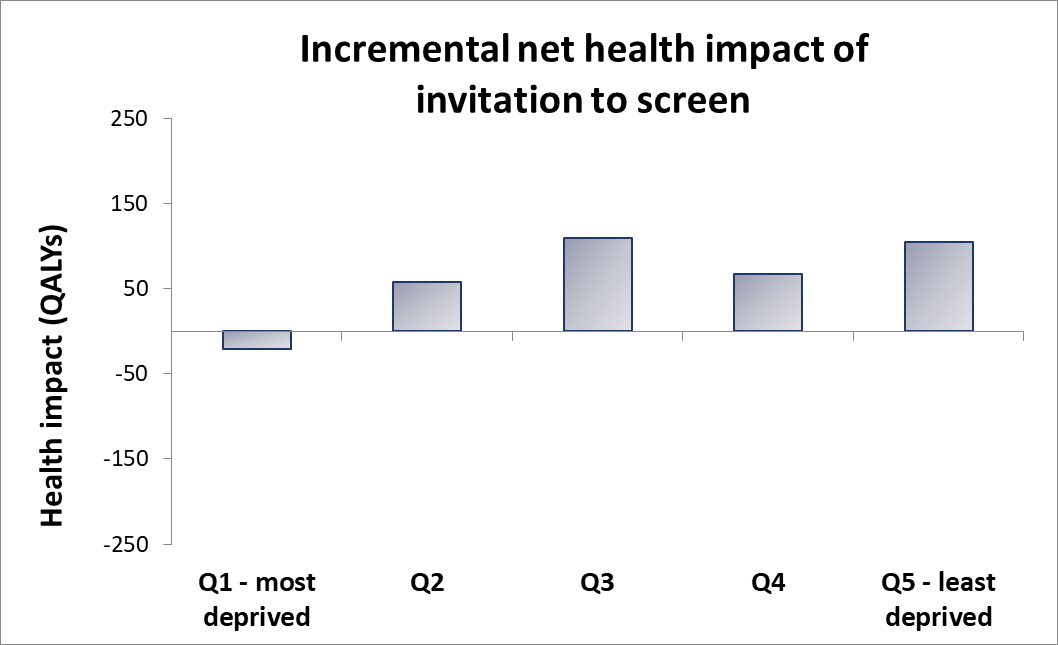


**Figure S3.A: Incremental net health impact for males aged 65 years (scenario analysis 1)**

**
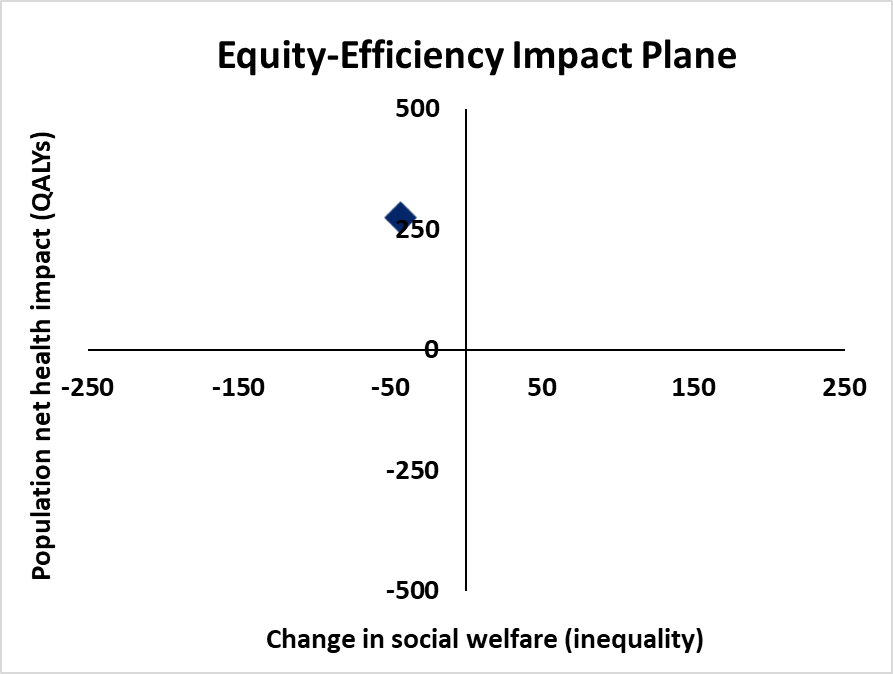
**

**Figure S3.B: Equity-impact plane at the population level (scenario analysis 1)**

**Supplementary Appendix 4**

Results of scenario analysis under a ‘levelling up’ best-case scenario for screening access, uptake and delivery.


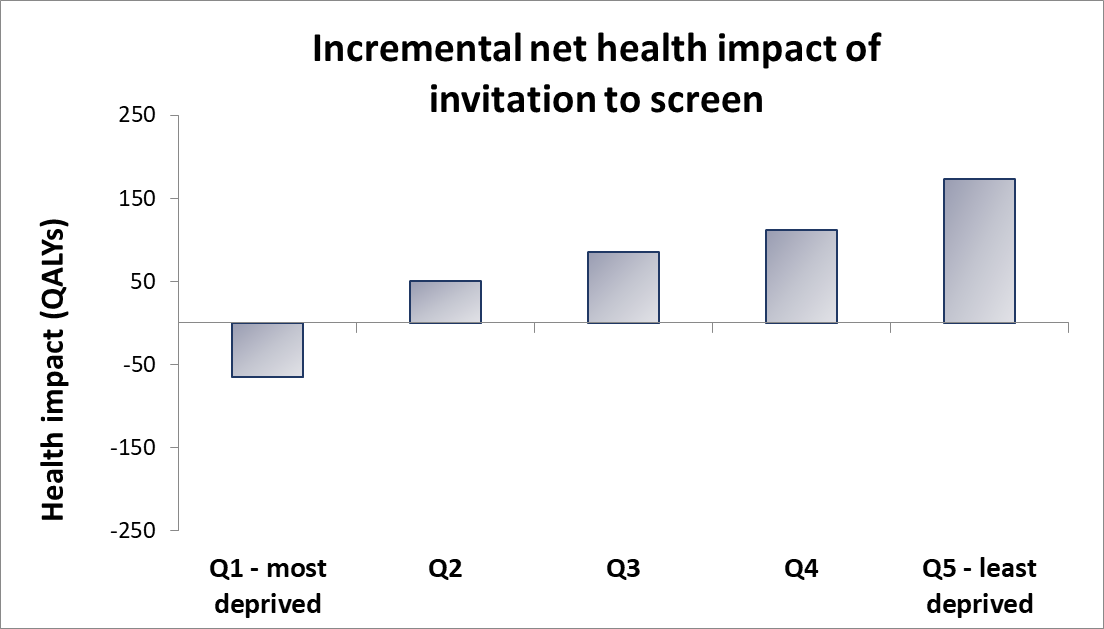


**Figure S4.A: Incremental net health impact for males aged 65 years (scenario analysis 2)**

**
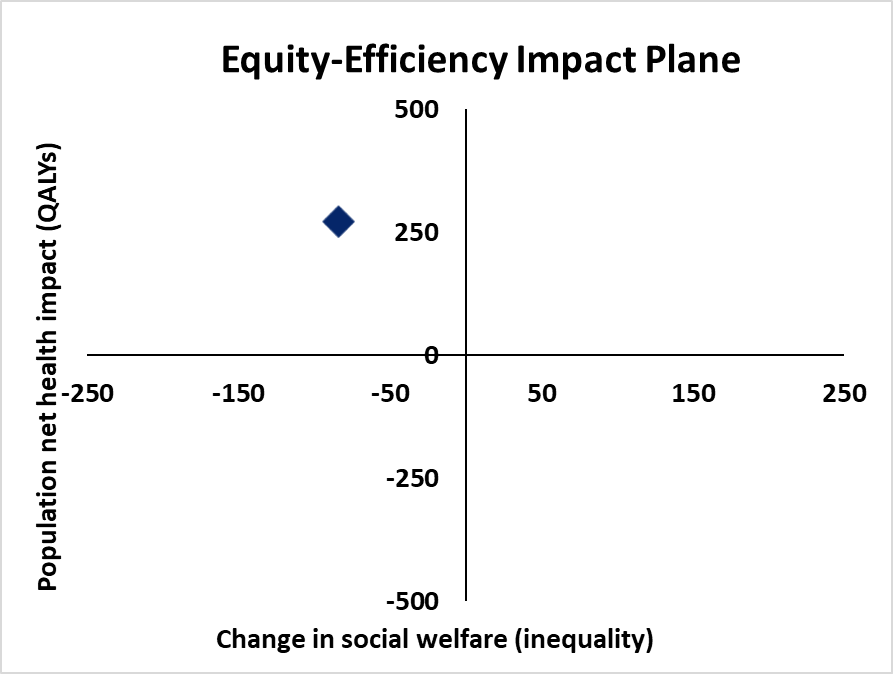
**

**Figure S4.B: Equity-impact plane at the population level (scenario analysis 2)**

**References**

1. Thompson SG, Bown MJ, Glover MJ, Jones E, Masconi KL, Michaels JA, et al. Screening women aged 65 years or over for abdominal aortic aneurysm: A modelling study and health economic evaluation. Health Technol Assess. 2018;22(43):V–141.

2. Office for National Statistics. Number of death registrations and population estimates by sex, single year of age and Index of Multiple Deprivation (IMD) Decile, England and Wales [Internet]. 2019 [cited 2023 Mar 12]. Available from: https://www.ons.gov.uk/peoplepopulationandcommunity/birthsdeathsandmarriages/deaths/adhocs/12413deathregistrationsandpopulationsbyindexofmultipledeprivationimddecileenglandandwales2019
